# Cartilage organoids: an emerging platform for novel osteoarthritis therapies

**DOI:** 10.3389/fcell.2025.1668766

**Published:** 2025-12-12

**Authors:** Bimin Gao, Zecai Chen, Yufeng Long, Feng He, Donghao Gan, Weihong Yi, Guozhi Xiao, Jiangdong Ren, Lei Qin

**Affiliations:** 1 Orthopaedic Innovation Laboratory, Shenzhen Nanshan People’s Hospital, The Affiliated Nanshan Hospital of Shenzhen University, Shenzhen, Guangdong, China; 2 Department of Osteoarthropathy, Shenzhen Nanshan People’s Hospital, Shenzhen, Guangdong, China; 3 Department of Spine Surgery, Shenzhen Nanshan People’s Hospital, Shenzhen, Guangdong, China; 4 Department of Orthopaedics and Rehabilitation, Yale University School of Medicine, New Haven, CT, United States; 5 Department of Biochemistry, Homeostatic Medicine Institute, School of Medicine, Guangdong Provincial Key Laboratory of Cell Microenvironment and Disease Research, Shenzhen Key Laboratory of Cell Microenvironment, Southern University of Science and Technology, Shenzhen, Guangdong, China

**Keywords:** osteoarthritis, cartilage organoids, drug screening, personalized therapies, in vitro models

## Abstract

Osteoarthritis (OA) is a chronic, multifactorial joint disorder characterized by the progressive degeneration and dysfunction of various joint tissues. Current treatments primarily focus on symptom management, offering limited success in halting cartilage degradation or repairing damaged tissues. Consequently, there is a pressing need for innovative therapeutic strategies aimed at cartilage regeneration and structural repair. Over the past 2 decades, cartilage organoids have emerged as a promising alternative for OA treatment. Due to their unique regenerative properties, cartilage organoids provide a versatile platform for various applications in OA research and therapy, including *in vitro* disease modeling, drug screening, regenerative medicine, and biomechanical studies. This review summarizes current research progress and insights into OA pathogenesis and therapeutic approaches, explores the development of cartilage organoid technologies with a focus on organoid constructions and different methodologies, and discusses the future applications of cartilage organoids as essential *in vitro* models for drug screening and personalized therapies for OA studies and treatment.

## 1 Introduction

Osteoarthritis (OA) is a chronic whole joint disorder characterized by the degeneration and malfunction of various parts of joint tissues, including the wear and tear of articular cartilage, the formation of osteophytes, and inflammatory responses in the synovial membrane and other tissues surrounding the joint (Glyn-Jones et al., 2015; Hügle and Geurts, 2017). OA can be found in all joint structures, including knee joints, hip joints, shoulder joints, and temporomandibular joint (TMJ), etc. (Coaccioli et al., 2022). OA causes joint pain, swelling and stiffness. In severe cases, OA can limit mobility and even disability (Abramoff and Caldera, 2020). OA is a common regenerative disease among elderly and the number of patients with OA worldwide had reached 595 million in 2020 (Collaborators, 2023). It is estimated that by 2050, the number of global patients with knee OA will continue to increase to 642 million (Collaborators, 2023; Safiri et al., 2020). The rising prevalence of OA, driven by global demographic aging, poses a substantial medical and economic burden on individuals and represents a growing public health challenge worldwide.

Currently, great efforts and progresses have been made to decipher the underlying mechanism of OA. However, the specific challenge of OA include two aspects: the precise molecular mechanism of the pathogenesis of OA is incompletely defined and the existing treatment methods for OA mainly alleviate symptoms which hardly achieves satisfactory results. Therefore, innovative therapies are needed for OA treatment. In response to this challenge, cartilage organoids, as a powerful tool, have been developed to simulate the microenvironment of articular cartilage, reproduce the occurrence and development process of OA, and utilize in potential drug screening. This emerging *in vitro* approach provides a unique platform for in-depth exploration of the disease mechanism, screening of and evaluation of the effectiveness of new therapies. Utilizing cartilage organoids for basic research and therapy exploration is expected to bring new hope for ultimately overcoming OA (Maharjan et al., 2024). In this review, we summarized our current understanding of OA pathogenesis and treatment, highlighted the construction of cartilage organoids, and discussed their current applications and future directions in OA treatment.

## 2 OA pathogenesis and current treatments

**FIGURE 1:**
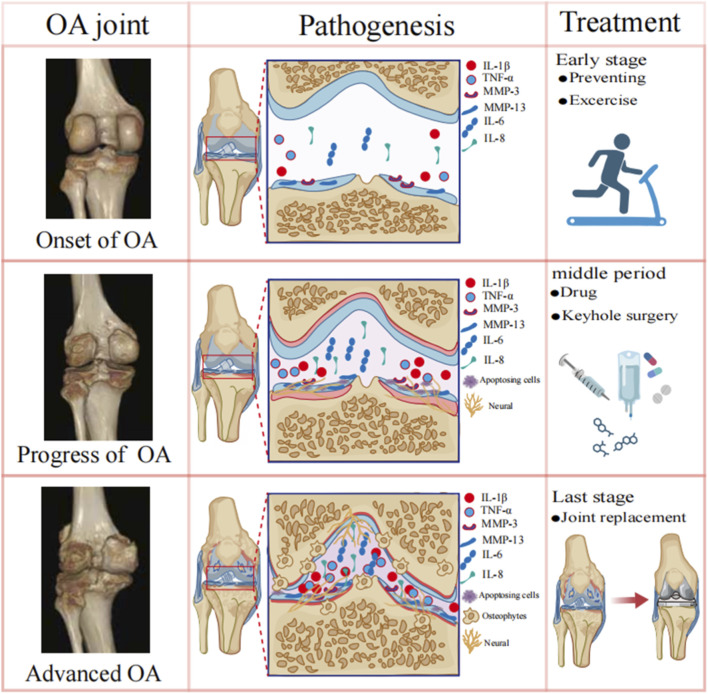
OA pathogenesis and current treatments. At the early stage, mild wear and roughness cause chondrocyte cell damage accompanied with a mild narrowing of the joint space. Inflammatory mediators (IL-1, TNF-α) released by damaged chondrocytes further activate macrophages to release pro-inflammatory factors (IL-1β and TNF-α) in the joint environment. Meanwhile, the proliferation of nerve endings in the joints increases so as their sensitivity. During this stage, patients are suggested with basic treatment, such as weight control, exercise therapy, and physical therapy. At the progress stage, the chondrocyte apoptosis is an important pathological process and the continuous nerve innervation into multiple joint tissues, thereby affecting joint pain and function. During this moderate stage of OA, patients can consider using painkillers, minimally invasive interventional procedures and intra-articular injections of PRP. In the advanced stage, new bone grows at the edges of cartilage, joint capsule and ligament junctions, forming osteophytes. When the OA of the patient has reached a severe stage and the doctor mainly adopts joint replacement surgery.

OA is a complex pathological process driven by multiple factors, involving the cellular apoptosis of chondrocytes, remodeling of subchondral bone, muscle weakness, inflammation, and interaction between neuro-immunity (Umoh and de Oliveira, 2024; Guilak et al., 2018; Yao et al., 2023) (Figure 1).

At the early stage, the X-ray film shows a mild narrowing of the joint space with obvious small osteophytes. Mild wear and roughness cause cell damage of chondrocytes, releasing inflammatory mediators, such as interleukin-1 (IL-1), tumor necrosis factor-α (TNF-α) and matrix metalloproteinase (MMP), into the joint environment (Woodell-May and Sommerfeld, 2020; Chen et al., 2020a). After the synovial membrane is stimulated by these inflammatory factors, the activated macrophages in the synovial membrane regulate the chondral microenvironment by secreting pro-inflammatory factors such as IL-1β and TNF-α, triggering systemic inflammation and activating NF-κB and MAPK pathways in chondrocytes, resulting in excessive production of degradation enzymes like MMP-3, MMP-13 and ADAMTS-5 by chondrocytes, thereby causing the degradation of collagenous extracellular matrix (ECM) (Mantovani et al., 2004; Lopes et al., 2017; Zhang et al., 2019; Shibo et al., 2024). Meanwhile, the levels of chemokines such as IL-6 and IL-8 in synovial fluid increase, forming a chronic inflammatory microenvironment (Samavedi et al., 2017). During the progress stage, X-ray films show definite narrowing of the joint space, moderate osteophytes, mild hardening of the subchondral bone, and possible bony deformity of the knee joint. The progressed hard bone changes are ly resulted from the increases in reactive bone formation. During OA progress, the chondrocyte apoptosis is an important pathological process. Reported studies indicated that some cytokines such as IL-1, TNF-α and TGF-β2, and the p38 MAPKs pathway and the Fas/FasL pathway link to chondrocyte apoptosis in OA pathogenesis (Hwang and Kim, 2015; Fazio et al., 2024; Sun et al., 2020). In the advanced stage, x-ray films show severe joint space stenosis, a large number of osteophytes, obvious subchondral bone sclerosis, and obvious bony deformity of the knee joint. During this stage, new bone grows at the edges of the cartilage, joint capsule and ligament junctions, forming osteophytes. This development of subchondral bone sclerosis and osteophytes is driven by the imbalance of Wnt/β-catenin and RANK/RANKL signaling pathways, which may gradually lead to overall joint dysfunction (Kovács and Nagy, 2019; Hongbo Ai et al., 2025). Moreover, abnormal loading promotes bone formation through the TGF-β/Smad pathway, resulting in excessive mineralization and a decrease in the mechanical stability of subchondral bone (Ting-Hsuan Wang et al., 2024).

During the OA pathogenesis, the pain is highly associated with nerve innervation and synovium inflammation in the affected joints. The joint is a highly innervated organ. In humans, the joint branches of the tibial nerve that innervate the posterior capsule of the knee joint contain 70%–80% unmyelinated C-fibres and sympathetic nerves. Pain receptors are widely distributed in multiple tissues such as the joint capsule, ligaments, periosteum, meniscus, subchondral bone, and synovium (Malfait and Schnitzer, 2013). Recent reported showed that netrin-1, an axon-guiding factor, may play essential roles in OA pain and OA progress. During OA progress, the metabolism of the subchondral bone altered and osteoclasts enhance the secretion of molecular netrin-1 (Zhu et al., 2019; Suri et al., 2007). Netrin-1 contribute to OA pain by two distinct approaches. On one hand, netrin-1 induces angiogenesis by promoting the function of endothelial cells at the bone-cartilage junction. On the other hand, it directly stimulates the growth of sensory nerve axons through the netrin-1 receptor, which in turn leads to abnormal changes in the surrounding nerve distribution. These processes mediated by netrin-1 jointly drive the progression of OA and triggers pain in the degenerated joint. In addition to nerve innervation during the progression of OA, macrophages accumulate in the joints and synovium, leading to inflammation, pain and cartilage degradation. Studies showed that pro-inflammatory macrophages (M1 macrophages) not only directly release pro-inflammatory factors to stimulate the injured nerve endings, but also enhance the sensitivity of sensory neurons to prostaglandin E2 (PGE2) by secreting cytokines such as TNF-α, thereby promoting hyperalgesia (Zou et al., 2024). Furthermore, nerve growth factor (NGF) can increase joint pain by stimulating neuronal growth and enhancing the sensitivity of peripheral nociceptive neurons. Aso K quantified the histopathology of OA tissues, NGF expression, and neuropeptide-calcitonin gene-related peptide (CGRP) nerves in OA model rats induced by medial meniscus resection before and after the operation at different time points. Their results showed that NGF expression had tissue specificity and time dependence: it increased early in the synovium and then decreased, but continued to increase in the bone-cartilage channels and bone marrow. Correspondingly, the CGRP nerve innervation, which is a marker of sensory nerves, also increased along with NGF expression, confirming that NGF is the core molecule driving the growth of joint nerves and pain associated with OA (Aso et al., 2022).

The therapeutic aim of OA treatment is to alleviate the pain symptoms, protect joint structure, and maintain or improve joint function. Depending on the different disease stages and patients’ conditions, clinical therapeutic treatment varies (Figure 1). At the early onset of OA, patients are suggested with basic treatment, such as weight control, exercise therapy, and physical therapy (Van Doormaal et al., 2020). During the moderate stage of OA, patients experience cartilage wear, narrowing of joint space and obvious osteophytes. If there is mild pain, one can consider using painkillers such as non-steroidal anti-inflammatory drugs, anti-anxiety and anti-depressants, anti-inflammatory drugs, and Chinese patent medicines, etc. (Hermann and Muller-Ladner, 2018; Wang et al., 2018; Khan et al., 2025). Moreover, intra-articular injections of hyaluronic acid and platelet-rich plasma (PRP) can be used as an additional treatment for OA patient to lubricate the joints and reduce the pain (Du et al., 2025; Sewell and Ö, 2022; Karaborklu et al., 2024). If these painkilling strategies cannot reach patients’ requirement, minimally invasive interventional procedures will be applied to alleviate symptoms. The common minimally invasive surgeries for treating OA include arthroscopy, unicompartmental knee arthroplasty (UKA), and high tibial osteotomy (HTO). Arthroscopy helps delay the progression of the disease by repairing the meniscus and ligaments (Yang et al., 2023a). UKA preserves the lateral compartment, anterior and posterior cruciate ligaments, and the patellofemoral joint, allowing it to bear the natural biomechanics of the knee joint (Lim et al., 2014). HTO reduces cartilage wear by correcting the lower limb alignment. These surgeries all have the advantages of minimal trauma and quick recovery (Liu et al., 2019). When OA has reached a severe stage and the patient’s joint movement becomes extremely painful with limited joint functions, the doctor mainly adopts joint replacement surgery (Anne, 2015).

In short summary, the current treatments for OA only relieve symptoms and the specific challenges remains, including the detailed molecular regulation and precise tissue-specific pathology during OA progress. Moreover, the current OA studies widely utilized traditional animal models, which have certain limitations such as high cost, long modeling time, ethical controversies and limitation to fully recapture human OA pathology. Therefore, there is an urgent need for innovative therapies to achieve cartilage regeneration for OA therapy.

## 3 Cartilage organoids

The development of tissue culture technology has been demonstrated with significant scientific potential as *in vitro* models to explore OA pathogenesis. Tissue culture helps to replicate tissues and organs *in vitro*, thereby facilitating drug screening and research on disease mechanisms. In general, two-dimensional (2D) and three-dimensional (3D) culture techniques are applied to chondrocyte studies. Traditional 2D culture technology, which allows stem cells or adult cells to grow in a two-dimensional manner on the surface of a culture dish, is simple to operate and has been widely used as a mature technique. However, this cultivation method cannot maintain the original characteristics of cells over the long term, nor can it simulate the complex microenvironment and processes within the body, such as signal transduction and changes in spatial structure (Jensen and Teng, 2020). Compared to 2D culture, 3D culture technology simulates the three-dimensional environment inside the body and enables cell proliferation and differentiation. Its advantages include the ability to preserve the *in vivo* microenvironmen and the ability of being intuitive and controllable.

In 1907, H.V. Wilson’s observations reveal the self-organization and regeneration of isolated sponge cells, which lays the foundation for the development of organoid (Wilson, 1907). The concept of organoids can be traced back to 1946. Smith and Cochrane first described it as “cystic organoid teratoma” when conducting research on cystic teratoma (Smith and Cochrane, 1946). In 2009, Hans Clevers transplanted Lgr5 (+) intestinal adult stem cells from mice into a matrix gel and cultivated intestinal organoids. This was the first description of the process of generating organoids from adult stem cells, and this technique has now become the basis for various organ and cancer research as well as personalized medicine (Sato et al., 2009). Kuo CJ upgraded the organoids from a “tumor morphology simulator” to a “tumor immunotherapy testing platform,” and designed a Patient-Derived Organoids (PDO) system with an air-liquid interface (ALI): the lower layer is a collagen gel providing support, where the organoids absorb nutrients; the upper layer is exposed to the air and directly acquires sufficient oxygen. Thus, cells grow at the interface between gas and culture medium. This system provides a living *in vitro* model for the study of tumor immune microenvironment and personalized immunotherapy testing (Neal et al., 2018). These have laid a solid foundation for the research and application of organoids. In 2014, Lancaster and Knoblich systematically proposed the concept of organoids and defined organoids as “collections of organ-specific cells that develop from stem cells or organ-progenitor cells and are capable of self-organizing through cell division and spatially restricted line differentiation in a manner similar to *in vivo*” (Lancaster and Knoblich, 2014). In the early 21st century, with the rapid development of regenerative medicine and stem cell technology, the discovery of embryonic stem cells (ESCs) and induced pluripotent stem cells (iPSCs) has opened up new avenues for the research of organoids (Takahashi and Yamanaka, 2006; Oldershaw et al., 2010).

Organoids are self-assembling 3D tissues generated from pluripotent stem cells or adult cells, which have been recognized as a emerging powerful tool for developmental biology and disease modeling (Dutta and Clevers, 2017; Hu et al., 2018). Moreover, organoids are formed to replicate the cellular, structural and functional characteristics of a real organ, which have been developing rapidly to narrow the gap between two-dimensional cultivation and animal models (Chen et al., 2022; Corrò and Li, 2020; Takasato et al., 2016; Zhang et al., 2024a). In addition, organoids possess the characteristic of being constructed from multiple types of cells, which is suitable for *in vitro* studies of various tissues and organs, thus promoting drug discovery and disease modeling efforts (Sato et al., 2009; Rossi and Lutolf, 2018).

### 3.1 A brief history of cartilage organoids development

Organoid technology of successfully constructing functional organ models using stem cells or adult cells has laid a solid technical foundation for the subsequent development of cartilage organoids (Rossi and Lutolf, 2018; Yi et al., 2021). Cartilage organoids are the specific 3D cartilage-like tissues constructed through self-assembling stem cells or adult cells by *in vitro* tissue engineering technology, whose structure and function are similar to those of natural cartilage (Yuhao et al., 2023). Cartilage organoids have great potential in treating degenerative joint diseases such as OA and cartilage defects, and are expected to alleviate joint problems related to aging (Wang et al., 2024).

**FIGURE 2:**
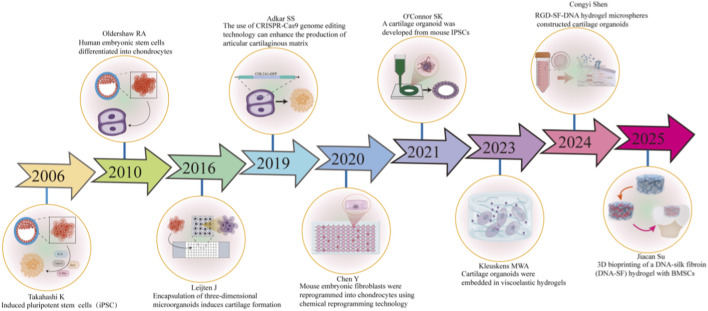
A brief development of cartilage organoids in the past 20 years. For the past twenty years, scientists from different research backgrounds systematically reported on the experimental process of developing new cartilage organoids. With the deep integration of materials science, bioengineering and clinical medicine, significant progress has been made in the technical construction and application expansion of cartilage organoids.

During the past 20 years, new techniques and applications have been gradually introduced into the development of multi-cellular and multifunctional cartilage organoids by researchers word-wide (Figure 2). In 2006, Takahashi K successfully induced iPSCs from mouse embryonic and adult fibroblast cells by introducing specific transcription factors, including Oct3/4, Sox2, c-Myc and Klf4 (Takahashi and Yamanaka, 2006). In 2010, Oldershaw RA reported an efficient and scalable approach that could direct the differentiation of human embryonic stem cells into chondrocytes (Oldershaw et al., 2010). In 2016, Leijten J induced the differentiation of soft growth plate cells and the formation of cartilage without growth factors by encapsulating 3D micro-organoids. This method can solve the problem of cartilage injury repair without the need for exogenous growth factors (Leijten et al., 2016). With the rapid development of gene editing technology, in 2019, Adkar SS utilized the CRISPR-Cas9 technology to achieve specific labeling and purification of chondrocytes, and engineered patient-specific tissue types, providing a pure cell source for subsequent cell therapy and basic research (Adkar et al., 2019). In 2020, Chen Y conducted 3D culture of chondrocytes induced by chemical mixtures and implanted them into defective articular cartilage. They found that these chondrocytes could promote cartilage repair, thereby verifying the biological functions of chemically induced chondrocytes (Chen et al., 2020b). In 2021, O'Connor SK reported a new type of chondrocyte organoid derived from mouse iPSCs provides a platform for studying the pathological interactions between bone and cartilage (O’Connor et al., 2021). In 2022, a novel stentless 3D cartilage regeneration technology was proposed by Huo Y, namely, cartilaginous organoids bioassembly (COBA), which can realize batch preparation of cartilaginous organoids. It greatly overcomes the limitations of traditional support materials (Yingying et al., 2024). In 2023, Kleuskens MWA cultivated cartilage organoids from non-degenerated cartilage cells and OA cartilage cells respectively, and embedded these organoids into viscoelastic hydrogels. These organoids derived from non-degenerated cartilages have greater potential in forming new cartilage (Kleuskens et al., 2023). As more disciplinary technologies are applied to the cartilage organogenesis technology, in 2024, Congyi Shen constructed cartilage organoids using silk fibroin-DNA hydrogel, which exhibits excellent biocompatibility and biointegration and serve as a new material option for cartilage regeneration (Shen et al., 2024). Earlier this year in 2025, the same team lead by JC Su developed a DNA-silk fibroin (DNA-SF) hydrogel sustained-release system (DSRGT), which enables controlled drug release through covalent grafting of glucosamine and TD-198946. Combined with 3D printing technology, it constructs millimeter-sized cartilage organoids. *In vitro* experiments showed that the organoids cultured for 4 weeks had the best transparent cartilage phenotype; after transplantation *in vivo*, the defect was repaired within 8 weeks through activating the MAPK signaling pathway to promote cartilage regeneration. Moreover, the gene expression profile of the regenerated cartilage was highly similar to that of healthy cartilage. This research provides an “organoid + sustained-release material” innovative strategy for cartilage defect repair, overcoming the problems of long traditional repair cycles and limited effects (Shen et al., 2025).

### 3.2 The construction of cartilage organoid

**FIGURE 3:**
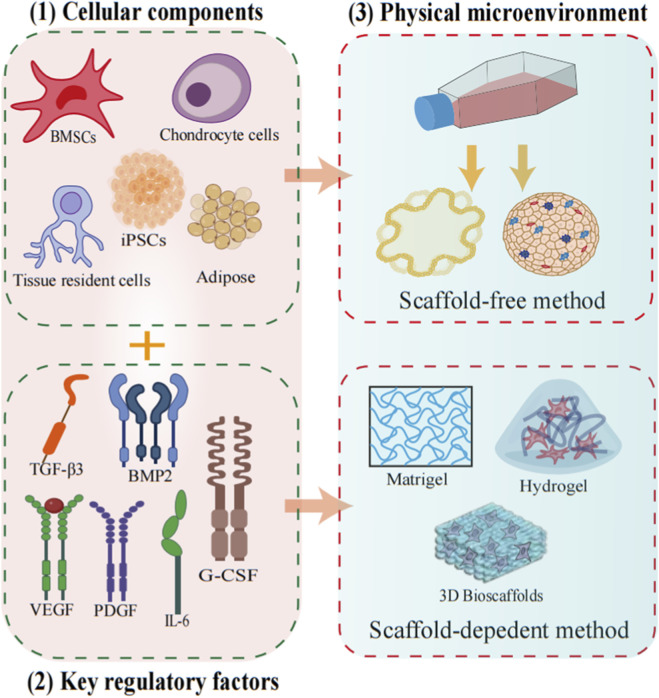
The construction of cartilage organoids. There are three fundamental and essential components: (1) cell component, (2) key regulatory factors and (3) physical microenvironment to achieve successful and functional cartilage organoids.

Successful cartilage organoids involve three fundamental and essential components, i.e., cellular components, key regulatory factors and the physical micro-environment (Figure 3). Importantly, cartilage organoids are not only the simple sum-up of these three components, but organic interaction as well as dynamic organization among different components.

#### 3.2.1 Cellular components

The cellular components for cartilage organoids were mainly primary chondrocytes and stem cells, which are originally derived from cartilage tissues or able to differentiated into chondrocytes *in vitro* (O’Connor et al., 2021; Tam et al., 2021; Li et al., 2022; Pittenger et al., 2019). Considering the different culture and treatment protocols for each cell types (Table 1), there is no reported data show structural or functional comparison among these cell sources in cartilage organoids construction.

**TABLE 1:** The constructions and applications of cartilage organoids.

Culture method	Cell sources	Key factors	Scaffolds/ Treatment	Outcomes and mechanisms	References
Scaffold-free method	Murine chondrocyte	Sox9	Chondrocytes cultured in the maintenance medium and supplemented with FGF2, BMP4, IGF1, leukemia suppressor factor, TGF-β1 and trumine for 3–4 weeks	SOX9/GLI1/BMPR1B/ Integrin α4/STAT3 ↓	Tassey et al. (2021)
	Murine iPSCs	TGF-β, BMP2	The cartilage organoids were cultured in chondrogenic media for 16 days, and in osteogenic media for 28 days	Alpl/Bglap/Col1a2/Ibsp/ Runx2/Sp7/Acan/Col2a1/ Prg4/Sox9 ↑ Type II and type VI collagen/s-GAG ↑ Type I and Type X collagen ↓	O’Connor et al. (2021)
	Monkeys’ iPSCs	BMP2, TGFβ1, GDF5	Chondrocytes were cultured in chondrogenesis medium for 2 weeks and then transferred to three-dimensional cultures to form cartilage particles	SIK3/PRG4 ↑	Abe et al. (2023)
	Human chondrocyte	TGFβ1, BMP2	Cartilage organoids were cultured in bone base organoid media, with RANKL added on day 5	COL2/ACAN ↑ Runx2/Sox9 ↓	Abraham et al. (2022)
	Human iPSCs	HbFGF, TGF-β1, BMP2, GDF5	iPSCs differentiated into chondrocytes for 14 days, and then chondrocyted self-assemble into chondroorganoids for 3 weeks	COL2A1/Sox9/VEGF/ MMP13 ↑ NANOG/OCT3/4/SOX2 ↓	Tam et al. (2021)
	Human iPSCs	Type II/type VI collagen, glycosaminoglycans, Sox9	The organoids were cultured for 24 days in chondrocyte experiment medium	Proteoglycans/type I collagen/type II collagen↓	Nakamura et al. (2021)
Scaffold-dependent method	Bovine chondrocyte	—	Chondrocytes were cultured the medium and the cells were encapsulated in hydrogel	Col2a1/Sox9 ↑ MMP-13/ADAMTS5/IL-1β ↓	Crispim and Ito (2021)
	Human MSCs	TGF-β1	Cell-adaptive supramolecular hydrogels support the rapid aggregation of MSCs to form cartilage organoids	Sox9/GAG/HIF-1α/LDHA/LDHB/MCT2/ MCT4/HAPLN147/ CRTAP/ACAN/TGFB2/ TGFBI/GDF/Type Ⅱ collagen ↑ Type I collagen/MMP13 ↓	Yang et al. (2025)
	Human umbilical cord MSCs	TGF-β, EGF FGF Gastrin	MSCs were inoculated onto microcarriers prepared using hyaluronic acid and hydroxyapatite, and then cultured in chondrogenic induction medium for 7 days to form cartilage organoids	Runx2/Col1a1/OCN ↑ Sox9 ↓	Yang et al. (2023b)
	human iPSCs and iPSCs	SOX9, COL2 COLX.	Cartilage microtissues derived from iPSCs combine with bone-forming “callus organoids” from HPDCS to form cartilage intermediate implants, resulting in the formation of cartilage during subcutaneous implantation	COLX/ALP/LEF1/ GREM1/FRZB ↑ Sox9 ↓	Hall et al. (2021)
	Human synovial MSCs and chondrocytes	TGFβ3 GDF5	SMSCs were nested in agarose micropores to form synovial MSC organoids and differentiated into cartilage microtissues in cartilage medium	TAOK1/GAG/COL I/COL II/SOX9/ACAN ↑ miR-24/HMGB1/p16^ink4a^ ↓	Sun et al. (2024)
	Human synovial MSCs	GDF5 TGF-β3	The combination of P16-siRNA encapsulated PLGA microspheres and the 3D culture of bioengineered cartilage-generated synovial MSC organoids ultimately created a P16-siRNA PLGA µS and SMSC organoid hydrogel-polymer composite scaffold (PPSOH)	SOX9/ACAN/COL2A1/ COMP/PRG4/ GLUT1 ↑ ADAMTS5/MMP13/MMP3/ COL10A1/Runx2/HMGB1/ CPT1a ↓	Yi et al. (2024)
	Human synovial MSCs and chondrocytes	TGF-β3, GDF5	Agarose microporous inserts are used to form a large number of uniformly sized synovial mesenchymal stromal cell organoids	ACAN/Sox9/Type II collagen/FOXC1/HIF3α ↑ MMP13/HIF1α ↓	Sun et al. (2021)
	naïve (undifferentiated) human BMSCs (hBMSCs)	—	Fibrin microbeads modified by covalent coating with hyaluronic acid (HyA-FMBs)	Type Ⅱ collagen/Lubricin ↑ type I collagen ↓	Kuznetsov et al. (2019)

Chondrocytes obtained from primary cell culture were used to construct tissue models that are closer to the structure of natural cartilage (Davies and Kuiper, 2019). Primary chondrocytes are easier and more cost-effective to build cartilage organoids, but the proliferation ability of such cells is limited. The construction of organoids is highly dependent on the regenerative potential and multi-directional differentiation ability of cultured cells, thus stem cells are the main cell sources for cartilage organoids.

Another main cell sources in cartilage organoids are adult stem cells, including pluripotent stem cells (PSCs) and mesenchymal stem cells (MSCs). PSCs, including embryonic stem cells (ESCs) and induced pluripotent stem cells (iPSCs), utilize their ability to proliferate indefinitely and differentiate into a variety of somatic cell types, and are wily used in the field of cartilage organoids (Bradley et al., 1984). ESCs are difficulty to obtain and encountered with ethical issues, therefore iPSCs are widely used in the construction of cartilage organoids studies (Takahashi and Yamanaka, 2006). iPSCs are reprogrammed cells obtained by reprogramming technology from somatic cells to pluripotent stem cells. It possesses the same pluripotency and unlimited proliferation potential as embryonic stem cells (Ikeda et al., 2004). The discovery of iPSCs has provided an alternative non-embryonic source. Their pluripotency is similar to that of embryonic stem cells and they can differentiate into various types of cells. For example, Tsumaki N transplanted the cartilage organoids derived from cynomolgus monkey iPS cells (cyiPSCs) to the cartilage defect in the knee joint of primate model. This experimental trail successfully achieved tissue repair and maintained for at least 4 months without triggering immune rejection. This study found that SIK3 regulates the expression of proteoglycan 4 (PRG4) protein, thereby endowing the transplanted cartilage with similar lubricating functions as the natural joint cartilage. This study demonstrated the feasibility of joint cartilage remodeling in primate models, laying the foundation for the development of cartilage defect therapies based on allogeneic pluripotent stem cells (Abe et al., 2023).

Adult stem cells, mainly mesenchymal stem cells (MSCs), also possess self-renewal and multi-directional differentiation characteristics, and due to their convenient sources, they have become a highly promising cell choice in tissue engineering (Arnold, 2017). MSCs can be extracted from bone marrow, fat or synovium to induce chondrogenic differentiation by TGF-β3 and BMP-2 signaling pathways (Neybecker et al., 2020; Shi et al., 2022; Qin et al., 2020). MSCs exhibit significant heterogeneity. Their differentiation is influenced by the source and culture conditions. Changes in phenotype during culture can lead to poor or abnormal cartilage differentiation (Pelttari et al., 2006). Chen Z constructed cartilage organoids using a single bone marrow-derived MSCs. By taking advantage of the natural vascularization gradient within the bone-cartilage tissue, he successfully guided the site-specific differentiation of the cartilage organoids, and achieved the regeneration of cartilage and bone. This study not only provides a new strategy for cartilage repair, but also offers important insights and directions for the field of tissue engineering (Chen et al., 2025).

#### 3.2.2 Key regulatory factors

The key regulatory factors for cartilage organoids include biochemical factors that influence the cellular behaviour in culture environment, such as cytokines, growth factors, and inflammatory factors (De Pieri and Zeugolis, 2021).

The TGF-β signaling pathway is involved in the processes of bone remodeling and cartilage repair, which plays an important role in the development and homeostasis maintenance of cartilage (Wu et al., 2024; Ye et al., 2022). Thus, TGF-β1, TGF-β3 and bone morphogenetic protein-2 (BMP-2) are key factors regulating cartilage differentiation. Their mechanism of action mainly involves activating the Smad signaling pathway, thereby promoting the differentiation of pluripotent stem cells like iPSCs into chondrocytes. TGF-β1 is a widely used growth factor that can promote the aggregation of chondrocyte progenitor cells, the differentiation of chondrocytes, and the maintenance of the chondrocyte phenotype. TGF-β3 is involved in regulating the survival, proliferation, migration and differentiation of chondrocytes (James et al., 2009; Du et al., 2023). Martin AR developed an electrospun cell-free fibrous hyaluronic acid scaffold, which was supplemented with factors that enhance cartilage repair: stromal cell-derived factor-1α (SDF-1α) and transforming growth factor-β3 (TGF-β3). It was found that the scaffold that only released TGF-β3 exhibited a higher cartilage formation ability compared to the scaffold that released only SDF-1α or the scaffold that released both factors. The regeneration of cartilage in the defects treated with the scaffold that released TGF-β3 was improved (Martin et al., 2021). In addition to TGF signaling, BMP-2 also plays a crucial role in the differentiation of chondrocytes and the maturation of the matrix. Studies showed that BMP-2 increases the matrix between chondrocytes and cartilage fibrous tissue, and promotes the ossification of chondrocytes (Kim et al., 2021). Dai K constructed cartilage organoids by using gelatin scaffolds loaded with BMP-2, which exhibited an immunosuppressive microenvironment during the stages of fibroblast proliferation and chondrocyte differentiation (Dai et al., 2023).

The cytokines used for the formation of cartilage organoids include insulin-like growtsh factor-1 (IGF-1), fibroblast growth factor (FGF), hepatocyte growth factor (HGF), platelet-derived growth factor (PDGF) and vascular endothelial growth factor (VEGF), etc. These cytokines can induce or stimulate the differentiation of chondrocytes (Fujii et al., 2018; Shen et al., 2022; Cui et al., 2022; Tonomura et al., 2020). IGF-1 inhibits NF-κB signaling transduction through specific PI3K/Akt and MAPK pathways, and prevents apoptosis by inhibiting reactive oxygen species (ROS) production, thereby promoting chondrocyte proliferation and matrix synthesis (Hossain et al., 2021; Jimi and Nakatomi, 2019). FGF is a protein family that plays a crucial role in various processes such as embryonic development, tissue regeneration, and wound healing (Kim et al., 2021). Foltz L added FGF8 at the final stage of inducing neural crest stem cells derived from human embryonic stem cells, which could promote the self-organization of cells into craniofacial cartilage organoids. These organoids not only possess the physical characteristics of cartilage (opaque and elastic), but also retain the key SOX9 protein, indicating that they have both the potential of neural crest origin and cartilage formation. This study revealed the specific signaling mechanism for human tissue differentiation and provided resources for subsequent studies on craniofacial development (Foltz et al., 2024). HGF is secreted by mesenchymal cells, which can enhance ECM production in chondrocytes and inhibit ROS and inflammation-induced cartilage damage (Ishibashi et al., 2016). PDGF is an important promoting factor for the proliferation of fibroblasts, osteoblasts and tendon cells, and it also has a positive effect on the regeneration and repair of cartilage tissue (Böhm et al., 2019). VEGF is a key signaling protein that mediates angiogenesis and plays a significant role in tissue repair. During wound healing, the body secretes large amounts of VEGF to stimulate the formation of new blood vessels, thereby providing support for the regeneration of damaged tissues. It is involved in the degeneration of articular cartilage and is a potential treatment option for patients with OA (Zhang and Xiao, 2016; Shang et al., 2018). Chen Z used agarose microarray culture to cultivate BMSC isolated from rabbit bone marrow and successfully constructed cartilage organoids by adding VEGF *in vitro*. The RNA sequencing results revealed that VEGF activated the Notch1/RUNX2 pathway, promoting the expression of genes related to angiogenesis and osteogenesis. This study confirmed that simulating the natural vascular gradient microenvironment is the key to regulating the specific differentiation of BMSCs, providing new ideas for clinical treatment (Chen et al., 2025).

#### 3.2.3 Physical microenvironment

Besides these biochemical factors listed above, the physical microenvironment, including mechanical stimulation, hypoxia environment, and scaffolds, play a crucial regulatory role in the formation of cartilage organoids.

Mechanical stimuli regulate chondrocytes differentiation and remodeling of ECM by influencing the dynamic microenvironment of articular cartilage in the body (Hoffmann et al., 2015). Nishino T applied a hinged external fixation device to rabbits for 6 months, and investigated the long-term effects of mechanical stimulation on cartilage repair after full-thickness defects. The results show that this mechanical stimulation promotes cartilage repair by increasing the content of type II collagen. This also confirms that weight-bearing has a positive effect on the quality of cartilage (Nishino et al., 2010). Furthermore, it has been proven that a low-oxygen environment is also conducive to the formation of cartilage organoids. Cartilage tissue has no blood vessels and its nutrition relies on diffusion. Hypoxia in the center of the growth plate will activate the HIF-1α pathway. Therefore, introducing hypoxia and HIF-1α signals helps to generate cartilage organ-like structures (Stegen et al., 2019). Yang B designed an adaptive supramolecular hydrogel of human bone marrow MSCs, which can adapt to the cell morphology and promote the rapid formation of large multi-cellular cartilage organoids. The establishment of the hypoxia microenvironment further promoted the formation of chondrocyte organoids. The research has found that the hypoxic microenvironment established within the chondrocyte organoids in the hydrogel can alter the morphology and function of mitochondria (Yang et al., 2025).

In addition to mechanical forces and hypoxia environment, cartilage organoids are normally constructed with or without scaffolds. Cartilage organoids frequently do not require the employment of carriers or scaffolds. Based on whether 3D scaffold materials are used, cartilage organoids can be classified into two categories: scaffold-free method and scaffold-dependent method (Table 1) (Yuhao et al., 2023).

##### 3.2.3.1 Scaffold-free method

The scaffold-free method is also called scaffold-free self-assembly method, in which cells autonomously forms 3D structures through intercellular interactions. Due to its simplicity and repeatability, it is the common culture method for cartilage organoids for drug development and high-throughput screening. Because the cellular aggregates are closer to the natural developmental process and can better simulate the environment of real tissues, this method is a favorable physiological model suitable for studying tissue development (Liu et al., 2024).

The scaffold-free methods include droplet culture, microfluidic chips and liquid overlay technique (LOT) (Velasc et al., 2020). Droplet culture is carried out by suspending cells on the cover of the culture dish, allowing the cells to freely aggregate and form 3D structures without adhesion (Qi et al., 2024). Inigo Martinez generated 3D structures without scaffolds using chondrocytes expanded in suspension method *in vitro*. All the culture conditions allowed the formation of 3D spheres. This solid 3D tissue formed by autologous cells is as an ideal alternative for autologous chondrocyte transplantation, providing a new treatment strategy for cartilage repair (Martinez et al., 2008). Moreover, microfluidics precisely controls the micro-environment for cell culture through the utilization of fluidic channels and pore structures (Li et al., 2024). For example, Shen C have developed a new type of RGD-SF-DNA hydrogel microsphere (RSD-MS) using microfluidics and self-assembly techniques, as a precursor for cartilage organoids (Shen et al., 2024). This method could promote the proliferation, adhesion and chondrogenic differentiation of BMSCs *in vitro*, and significantly promoted cartilage regeneration *in vivo*. In addition, LOT method enables cells to spontaneously aggregate into spherical structures within a short period of time by weakening the adhesion between cells and the surface of the culture material (Costa et al., 2014). This LOT method provides a new technological platform for studying intercellular communication and is suitable for experiments that require precise reproduction of the *in vivo* environment.

Compared to the other two methods, the LOT method produces reproducible spheres for multiple cell lines. This method has the advantages of simple operation, low cost, strong scalability, and is easy to be deployed in a conventional laboratory. It can also meet the requirements of high-throughput screening. In the LOT method, the formation of the spheres depends on the self-aggregation ability of the cells. By optimizing the culture medium or adding matrices, the aggregation effect of the spheres can be effectively improved (Jubelin et al., 2023).

##### 3.2.3.2 Scaffold-dependent method

The scaffold used for constructing cartilage organoids is a 3D porous matrix that provides a specific microenvironment to facilitate cartilage repair and regeneration. The ideal cartilage culture scaffold should not only possess appropriate physical properties (such as stiffness, flexibility, structure, porosity, permeability and biointegration), but also be capable of effectively promoting the adhesion, proliferation, aggregation and differentiation of cells. For instance, materials such as hydrogel, agarose, collagen, hyaluronic acid or polylactic acid (PLA) can provide the necessary physical support for the cultured cells, helping to achieve cell adhesion and matrix deposition (Chen et al., 2023; Teng et al., 2021; Fanrong et al., 2020; Jingyuan et al., 2022).

Hydrogels, characterized by high water-content, biocompatibility and tunable mechanical properties, are the most widely used biological materials in organoid research. Their three-dimensional network structure can simulate the production of ECM, providing an appropriate physical support and signal transmission for organoids. The high modifiable properties of hydrogels enable their combination with other *in vitro* approaches in the construction of cartilage organoids. For example, Yi Wang combined 3D bioprinting of hydrogels with siRNA technology to successfully construct a human synovial mesenchymal stromal cell (SMSC) organoid hydrogel (PSOH) that incorporates p16-siRNA. This organoid scaffold can effectively promote cartilage regeneration by up-regulating the expression of cartilage formation markers (such as SOX9, ACAN), while down-regulating the expression of decomposition and aging markers related to OA (such as MMP13, P16). Both *in vitro* and *in vivo* experiments have confirmed that PSOH has strong anti-aging and cartilage repair capabilities, providing a new strategy for treating cartilage defects and OA (Yi et al., 2024). Moreover, Chen Z used photolithography molds to fabricate agarose hydrogel scaffolds and utilized BMSCs to construct cartilage organoids *in vitro*. This method was the first to achieve a three-layer biomimetic structure of cartilage - calcified cartilage - subchondral bone, providing a new idea for clinical repair of bone and cartilage defects (Chen et al., 2025). In addition, Kuznetsov attached the naïve human bone MSCs (BMSCs) to the fibrin microbead scaffolds coated with hyaluronic acid and subcutaneously implanted into mice with low immune function. When the BMSCs were connected to the covalently coated hyaluronic acid fibrin microbeads (FMBs), they could efficiently form stable and non-giant transparent cartilaginous tissues. This breakthrough has opened up new avenues for the regeneration and repair of damaged joint cartilage and the *in vivo* modeling of human cartilage diseases (Kuznetsov et al., 2019).

Different cartilage tissue engineering methods may choose scaffolds based on their research purposes. Scaffold-free method of cartilage organoids is closer to the natural state and is easier to operate, but its mechanical strength and stability are relatively weak. Scaffold-dependent method of cartilage organoids is suitable for the construction of cartilage tissues with high mechanical strength and complex structures, but the operation is complex and the biological compatibility of the scaffold materials needs to be paid attention to. The choice of construction method should be comprehensively considered in light of the specific experimental requirements.

In summary, the construction of cartilage organoids aims to simulate the complex structure and biological functions of natural cartilage through the synergistic effects of cell sources, 3D biomaterial scaffolds, biological activators, and dynamic physical microenvironments.

## 4 Current applications of cartilage organoids in OA treatment

**FIGURE 4:**
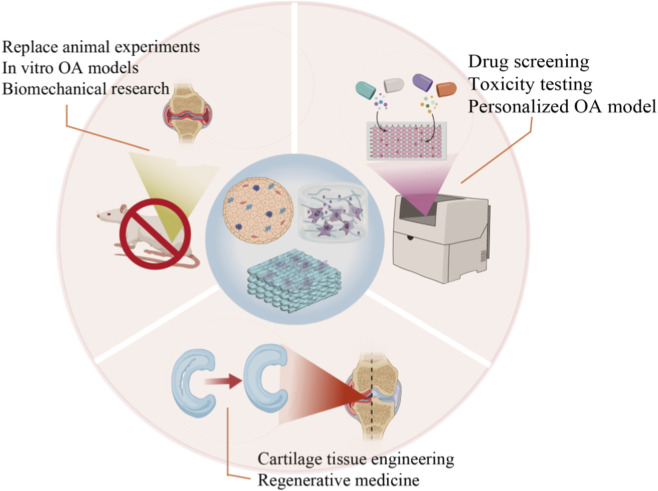
The applications of cartilage organoids in OA pathogenesis and treatment. Cartilage organoids, with their highly biomimetic characteristics, provide an innovative platform for multifunctional applications in OA pathogenesis and treatment. These applications include in vitro disease models and biomechanical studies for OA pathogenesis, drug screening, regenerative medicine and personalized medicine for OA treatment.

The pathogenesis of OA involves complex multi-factor interactions. Cartilage organoids, with their highly biomimetic characteristics, provide an innovative platform for multifunctional applications in OA pathogenesis and treatment, which includes *in vitro* disease models, drug screening, regenerative medicine and biomechanical studies (Aikang et al., 2025) (Figure 4).

### 4.1 Establishing OA disease model

The exploration of the mechanism of OA includes various *in vivo* and *in vitro* models. Although animal OA models are widely used in current research, there are several unsolved limitations associated with these *in vivo* models. Firstly, there are differences in anatomy, physiology and mechanics between animal and human cartilages, making it difficult for the models to fully simulate human conditions. Secondly, some models (such as spontaneous models) have long cycles and high costs, while traditional surgical modeling is difficult to precisely control the conditions and also cannot simulate traumatic osteoarthritis. On the contrary, cartilage organoids, especially derived from human samples, can simulate the pathological process of OA and provides a reliable model that is close to the real physiological state in human.

To establish OA disease model, two approaches are commonly used. One approach is to culture defected chondrocytes derived from OA patients. Utilizing patient-derived organoids can provide a deeper understanding of genetic heterogeneity among individuals, thereby supporting personalized medicine. The other approach is to induce cellular defects or chondrocyte degeneration *in vitro* with genetic modification or cultured with key factors, such as inflammatory factors IL-1β and TNF-α (Muzhe et al., 2022). For example, Dönges L utilized healthy donor’s human bone marrow-derived mesenchymal stromal cells (hBMSC) and first carried out 14 days of chondrogenic differentiation, followed by 14 days of hypertrophic differentiation. During this process, inflammatory factors such as IL-1β, IL-6 and TNFα were added. Successfully, a cartilage organoid model of OA was constructed. Subsequent marker staining, secretion protein and gene expression analyses all indicated that this model exhibited typical characteristics of OA cartilage degeneration, laying a solid model foundation for related research (Dönges et al., 2024). Moreover, other factors such as IL-1Ra (interleukin-1 receptor antagonist) can effectively improve the pathological features of OA in cartilage organoids (Laura Dönges et al., 2024). This highlights the significant value of human OA chondrocyte organoids in revealing the mechanisms of cartilage degeneration and in screening potential therapies. Furthermore, Tassey J cultivated chondrocytes in a 3D environment to form cartilage microspheres. After treating them with pro-inflammatory cytokine OSM for 3 weeks, cartilage organoids were formed. This model can simulate the characteristics of osteoarthritis and provides a new tool for understanding the complex behaviors of chondrocytes and how they are influenced by their surrounding environment (Tassey et al., 2021). In addition to investigate the pathogenic mutations in families with early-onset osteoarthritis, van Hoolwerff M used the CRISPR-Cas9 gene editing technology to introduce the highly influential FN1 mutation into human pluripotent stem cells, and constructed an *in vitro* cartilage organoids. The results showed that the FN1 mutation significantly reduced the chondrogenic potential of chondrocytes and the deposition of new cartilage. This suggests that the pathogenic mechanism of the FN1 mutation may be related to the decreased ability of fibronectin to bind to type II collagen of the ECM. This study used *in vitro* cartilage organoids to explore the potential pathological pathways of osteoarthritis and is expected to promote the development of new therapies (Van Hoolwerff et al., 2021).

In short summary, cartilage organoids with clinical patient-derived cells and *in vitro* pharmaceutical treatment provide essential *in vitro* OA models, which is close to the real physiological states with high ability to simulate the pathological process of osteoarthritis for detailed molecular studies.

### 4.2 Drug screening

The traditional clinical drug development process is lengthy and costly. Due to the differences between animal experiment results and human responses, the success rate is relatively low. Cartilage organoids can directly use patient cells for drug screening, bypassing some of the traditional experiments and significantly shortening the research and development cycle (Lin et al., 2023). Moreover, using cartilage organoids to screen OA drugs can enhance the accuracy of drug efficacy assessment, support the development of personalized therapies for osteoarthritis, and solve the problem of insufficient predictability of traditional models (Boone et al., 2025).

For example, Wei X’s team has constructed a cartilage organoid based on human expanded pluripotent stem cells (hEPSCs), and used the dual-reporter genes COL2A1^mCherry^ and COL10A1^eGFP^ to monitor the processes of cartilage formation and hypertrophy in real time. Through high-throughput screening of a library of over 2,000 FDA-approved marketed drugs, it was revealed that the α-adrenergic receptor (α-AR) inhibitor phentolamine can induce cartilage formation and inhibit hypertrophy, while the α2-AR agonist inhibits cartilage formation and induces hypertrophy. The mechanism lies in that the α2-AR signaling pathway induces the production of cyclic guanosine monophosphate (cGMP)-dependent leukocyte protease inhibitor (SLPI), thereby causing the hypertrophic degeneration of cartilage. On the contrary, when SLPI is absent, cartilage organoids exhibit anti-degenerative capabilities, which is beneficial for the healing of large cartilage defects. Moreover, Boone I and colleges aim to conduct effective preclinical tests on drugs for osteoarthritis and anti-aging drugs (such as senolytics and senomorphics), and for this purpose, a 3D high-throughput human *in vitro* cartilage organ model has been developed as a drug testing platform (Wei et al., 2024). Together, these studies conducted drug screening using cartilage organoids, not only identifying potential therapeutic targets for cartilage regeneration, but also providing a platform that is not subject to ethical restrictions for human developmental studies.

### 4.3 Cartilage tissue engineering and regeneration

Cartilage tissue healing and regeneration is always being the research focus for OA treatment. Cartilage organoids have the advantages of mimicking the structure and function of natural cartilage, reducing the donor limitation and immune rejection issues. Moreover, cartilage organoids can be mass-produced through stem cell technology, which makes them efficient and feasible alternative approach. Therefore, cartilage organoids offer innovative strategies for cartilage tissue engineering and regenerative medicine (Burdis and Kelly, 2021).

Regenerative therapies for cartilage include traditional surgical procedures and cell-based therapies to advanced tissue engineering approaches utilizing scaffolds, growth factors, and bioprinting, which are continuously bringing new insights and therapeutic potential to the treatment of OA. For example, Zhu J currently developed a multifunctional self-assembling hydrogel that can locally release the anti-aging miR-29b-5p and simultaneously recruit endogenous synovial MSCs. By continuously delivering miRNA and guiding the differentiated proliferation of the recruited MSCs into chondrocytes, this strategy achieved effective cartilage repair and regeneration (Zhu et al., 2022). Moreover, Jiang H and colleges created cartilage extracellular matrix microcarriers (CEMMs) from pig cartilage by employing techniques of decellularization, wet grinding, and layered screening. Subsequently, they used the CEMMs to construct cartilage tissue spheres and verified their effectiveness in an SD (Sprague Dawley) rat model of cartilage defect. Micro-CT analysis revealed that the organoid group showed significant repair effects: the defect was filled more completely, the surface was smoother, and the regeneration of subchondral bone was better. This achievement not only demonstrated the feasibility of constructing organoids using cartilage-derived decellularized matrices, but also provided a new strategy for promoting cartilage regeneration (Jiang et al., 2025).

### 4.4 Biomechanical research

Cartilage tissue, as a type of connective tissue, has its own unique mechanical properties. The impact of mechanical stimulation on articular cartilage has received increasing attention. Healthy cartilage tissue is seen as a permeable, viscous-elastic materials, which consist two phases, i.e., a fluid phase and a solid phase (Eschweiler et al., 2021). Since cartilage contains mostly of water (up to 65%–85%), the principal component of its fluid phase is water. The solid phase of cartilage is mostly dependent by ECM. During the joint loading, the compressive load causes interstitial fluid pressure and leads to a fluid flow out of the ECM in cartilage. Whereas when the load is removed, the interstitial fluid flows back from the inner joint space in to the ECM of cartilage. Therefore, instead of Young’s modulus, the aggregate modulus (a measurement of the stiffness of the tissue at the equilibrium when all fluid flow has slowed down) is often used to describe cartilage tissue. The average aggregate modulus of cartilage is typically in the range of 0.5–0.9 MPa (Eschweiler et al., 2021; Chung et al., 2015).

With the optimization of cultivation conditions and the development of 3D culture techniques by using various biomaterials as scaffolds and modifying the cell composition, matrix secretion (such as type II collagen, proteoglycans), and mechanical properties, the biochemical and mechanical (Young’s modulus ≈ 1–2 MPa) of cartilage organoids are achieved similar to those of natural cartilage (Eyre and Wu, 1995; Zhou et al., 2023). To explore the potential mechanism of mechanical load in cartilage tissue engineering, Jia Y found that after a rigorous assessment of the effects of compression, fluid shear stress, hydrostatic pressure, and osmotic pressure on chondrocytes, mechanical stimulation did not have a negative impact on the survival rate and growth of the cells. However, only mechanical loads within a specific amplitude and time range are beneficial for the regeneration of articular cartilage, while maximum and supra-maximum mechanical loads are harmful to articular cartilage. This study significantly advances the development of cartilage tissue engineering by simulating the natural mechanical environment (Jia et al., 2023).

## 5 Challenges of cartilage organoid technology

Even though there are promising application prospects of cartilage organoids in the treatment of OA, remaining challenges limit the scope and effectiveness of cartilage organoids in clinical applications. The construction of cartilage organoids requires precise control of culture conditions, including the selection of biomaterials, the addition of cytokines, and the simulation of physical environments. The fine regulation of these conditions is crucial for the successful construction and functional maintenance of organoids (Hu et al., 2025; Bajic et al., 2020).

First, since OA is a whole joint disease, study with cartilage organoids only answer part of the question. Mature organoids need to replicate “Mini-joint” *in vitro* which contain multiple types of cells. However, different cells require different conditions for *in vitro* cultivation, which poses a challenge for the construction of multi-cellular organoids. Currently, single-cell organoid cultivation has become relatively mature, and dual-cell co-culture has also made progress. However, the construction of mature organoids containing multiple types of cells still requires overcoming the technical difficulties of multi-cellular co-culture and exploring compatible cultivation conditions. Furthermore, during the cultivation process, the internal cells may encounter problems of insufficient nutrient and oxygen supply. Due to the 3D nature of organoids, if they are too large (up to millimeter or centimeter levels), the internal cells are prone to being isolated from the external cells, resulting in a lack of sufficient nutrients and ultimately death. Therefore, how to effectively prevent cell aging and death during the construction process is an important challenge (He et al., 2022).

Second, the preparation and optimization of culture conditions or physical microenvironment is still a challenge in the construction of cartilage organoids for OA treatment. Among all the factors, the biocompatibility and mechanical properties of scaffolds still need to be optimized (Yi et al., 2021). On the one hand, ideal scaffold materials are difficult to obtain: natural materials have good biocompatibility but high processing costs, while synthetic materials are easy to process but lack cell-inducing properties. On the other hand, the structural design of the scaffold (such as porosity, mechanical properties) needs to precisely match the cartilage tissue, making it very difficult to achieve a complex structure with uniform and controllable degradation. Moreover, integrating bioactive molecules to promote cell behavior also faces challenges in effective delivery and maintaining activity (Faeed et al., 2024). Although the biochemical regulatory factors are powerful tools for construction cartilage organoids, their application faces multiple challenges. These factors have pleiotropic effects and complex interaction networks, and different factors have synergistic, antagonistic or overlapping effects. Therefore, it is difficult to precisely control their dosage and concentration. At the same time, different individuals or different animal models may have different responses to the factors, and the microenvironment in which the cells are located (such as ECM, co-cultured cell types, etc.) can significantly affect the effect of factors. These issues all require more basic research and clinical practice to explore.

Last but not least, there is a lack of unified standards in the production and validation of cartilage organoids. Independent studies reported diverse cell sources, culture conditions and treatment in the laboratory, which are validated mainly with *in vitro* methods including chondrocyte-specific mRNA and protein expression test, cartilage staining. However these *in vitro* validation neither provide good histological evidence of real cartilage, nor cartilage repair or regeneration trails *in vivo*. Moreover, to achieve clinical application, it is difficult to produce functional and fully structured cartilage organoids at a low cost, safely, and on a large scale. Therefore, it is necessary to develop efficient, scalable, and standardized production technologies that comply with Good Manufacturing Practice (GMP) to meet clinical and commercial needs (Desai and Gishto, 2015).

## 6 Conclusion and perspectives

The complex pathogenesis of OA continues to present significant challenges, leaving critical gaps in the existing body of research. While current pharmacological and surgical interventions offer some benefits to patients, a deeper understanding of the underlying mechanisms, as well as the development of novel therapeutic strategies, remains imperative. Cartilage organoids, with their biomimetic properties, adaptability, and efficiency, have the potential to revolutionize both fundamental research and therapeutic applications in OA.

With the fast development of new techniques, several groundbreaking technologies have been applied for further enhancement and expansion of cartilage organoids. For example, organ-on-a-chip is an engineered or micro-manufactured culture system that incorporates microfluidic channels, enabling it to naturally simulate the dynamic shear stress of *in vitro* culture, thereby enabling the simulation of organ functions (Zhang et al., 2024b). The dynamic integration of cartilage organoids and organ-on-a-chip will make great advantages of cell variability of organoids and device complexity of organ-on-a-chip, which shall bring new insights to clinical studies (Yimu et al., 2024). Moreover, micro-manufacturing and micro-fluidic technologies enable the controllable size of organoids, facilitating the construction of precisely sized organoids structures. These technologies can enhance the reproducibility of organoids, offer various environmental control and monitoring capabilities, and support large-scale production (Velasc et al., 2020; Shyam and Palaniappan, 2023). In addition, the tissue clearing technique enables high-resolution confocal imaging of 3D architectures of transparency tissues. Ni Y introduced an optimized solution for 3D analysis of bone tissue, achieving efficient visualization of hematopoietic and stromal cells in transparent bone samples, providing a reliable tool for studying rare cell populations and their spatial dynamics within organs (Ni et al., 2025). Hence, these technologies provide more accurate and efficient solutions for the basic research and clinical application of organoids.

By facilitating interdisciplinary integration of cutting-edge technologies, cartilage organoids not only provide new insights into the pathophysiology of OA but also hold promise for accelerating the translation of therapeutic innovations from preclinical studies to clinical practice.
